# Evolution of amorphous ruthenium nanoclusters into stepped truncated nano-pyramids on graphitic surfaces boosts hydrogen production from ammonia†

**DOI:** 10.1039/d4sc06382a

**Published:** 2025-01-09

**Authors:** Yifan Chen, Benjamin J. Young, Gazi N. Aliev, Apostolos Kordatos, Ilya Popov, Sadegh Ghaderzadeh, Thomas J. Liddy, William J. Cull, Emerson C. Kohlrausch, Andreas Weilhard, Graham J. Hutchings, Elena Besley, Wolfgang Theis, Jesum Alves Fernandes, Andrei N. Khlobystov

**Affiliations:** a School of Chemistry, University of Nottingham University Park NG7 2RD Nottingham UK jesum.alvesfernandes@nottingham.ac.uk andrei.khlobystov@nottingham.ac.uk; b Nanoscale Physics Research Laboratory, School of Physics and Astronomy, University of Birmingham Edgbaston B15 2TT UK w.theis@bham.ac.uk; c Max Planck Centre on the Fundamentals of Heterogeneous Catalysis FUNCAT, Cardiff Catalysis Institute, Translational Research Hub, Cardiff University Cardiff CF24 4HQ UK

## Abstract

Atomic-scale changes can significantly impact heterogeneous catalysis, yet their atomic mechanisms are challenging to establish using conventional analysis methods. By using identical location scanning transmission electron microscopy (IL-STEM), which provides quantitative information at the single-particle level, we investigated the mechanisms of atomic evolution of Ru nanoclusters during the ammonia decomposition reaction. Nanometre-sized disordered nanoclusters transform into truncated nano-pyramids with stepped edges, leading to increased hydrogen production from ammonia. IL-STEM imaging demonstrated coalescence and Ostwald ripening as mechanisms of nanocluster pyramidalization during the activation stage, with coalescence becoming the primary mechanism under the reaction conditions. Single Ru atoms, a co-product of the catalyst activation, become absorbed by the nano-pyramids, improving their atomic ordering. Ru nano-pyramids with a 2–3 nm^2^ footprint consisting of 3–5 atomic layers, ensure the maximum concentration of active sites necessary for the rate-determining step. Importantly, the growth of truncated pyramids typically does not exceed a footprint of approximately 4 nm^2^ even after 12 hours of the reaction, indicating their high stability and explaining ruthenium's superior activity on nanotextured graphitic carbon compared to other support materials. The structural evolution of nanometer-sized metal clusters with a large fraction of surface atoms is qualitatively different from traditional several-nm nanoparticles, where surface atoms are a minority, and it offers a blueprint for the design of active and sustainable catalysts necessary for hydrogen production from ammonia, which is becoming one of the critical reactions for net-zero technologies.

## Abstract 2

Atomic-scale changes can significantly impact heterogeneous catalysis, yet their atomic mechanisms are challenging to establish using conventional analysis methods.

## Graphical abstract

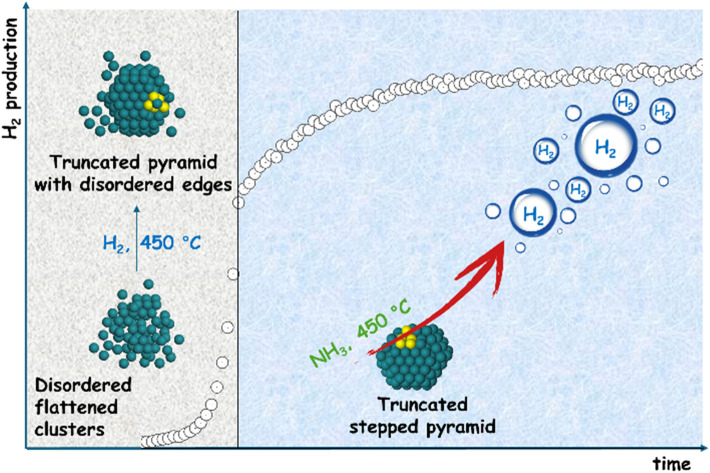

## Introduction

Heterogenous nano-catalysts are polydisperse materials with varying metal particles, with each particle possessing its unique shape, size, and structure, and hence its unique catalytic property. When we test the performance of a catalyst, the catalytic performances of individual particles are averaged. In the case of activity per gram of metal, the integral activity is divided by the total amount of metal in the catalyst. Therefore, for convenience, it is traditional to average the size of particles and correlate the average size with the macroscopic properties of the catalyst. For example, when the average size increases, the fraction of surface atoms decreases, which is expected to lead to reduced catalyst activity.

The situation is complicated by the fact that heterogeneous catalysts are not static materials. They undergo changes over time under reaction conditions, which are usually detrimental due to particle coarsening, surface poisoning, or metal leaching.^1^ Therefore, they have been extensively studied to understand how to avoid deactivation of catalysts.^2^ However, changes in the catalyst during the reaction can also be beneficial, which is discussed less often. These changes usually occur in a short period of time at the early stages of the reaction and are referred to as catalyst conditioning or activation. In some rare cases, these self-improvements of the catalyst continue over several hours through the reaction as observed in hydrogen production from water^3^ and from ammonia.^4^ The latter reaction is particularly topical because ammonia is gaining popularity as a zero-carbon energy vector.^5,6^ It is crucial to comprehend the catalyst evolution mechanism at the individual particle level, particularly for ruthenium, which is considered the most effective metal for NH_3_ decomposition, and can be supported on the surface of metal oxide,^7^ nitride,^4,8^ or carbon materials.^9,10^

Our study used identical location scanning transmission electron microscopy (IL-STEM) to examine changes in a Ru catalyst during the ammonia decomposition reaction. By tracking the evolution of individual nanoclusters in specific locations, we found that both Ostwald ripening and coalescence processes occur at a local scale. The major restructuring of Ru nanoclusters occurs due to the larger fraction of surface atoms in nanoclusters compared to traditional nanoparticles and the strong bonding of Ru to the carbon support. The number of atoms in each Ru nanocluster increases as the footprint and number of layers expand during H_2_ treatment. However, during the NH_3_ decomposition reaction, the footprint decreases while the number of atoms continues to grow, causing the nanoclusters to become taller and progressively pyramidal with stepped edges. These nanoscale changes are correlated with the increasing rate of hydrogen production from ammonia.

## Results and discussion

### Material preparation and catalytic performance

**Fig. 1:**
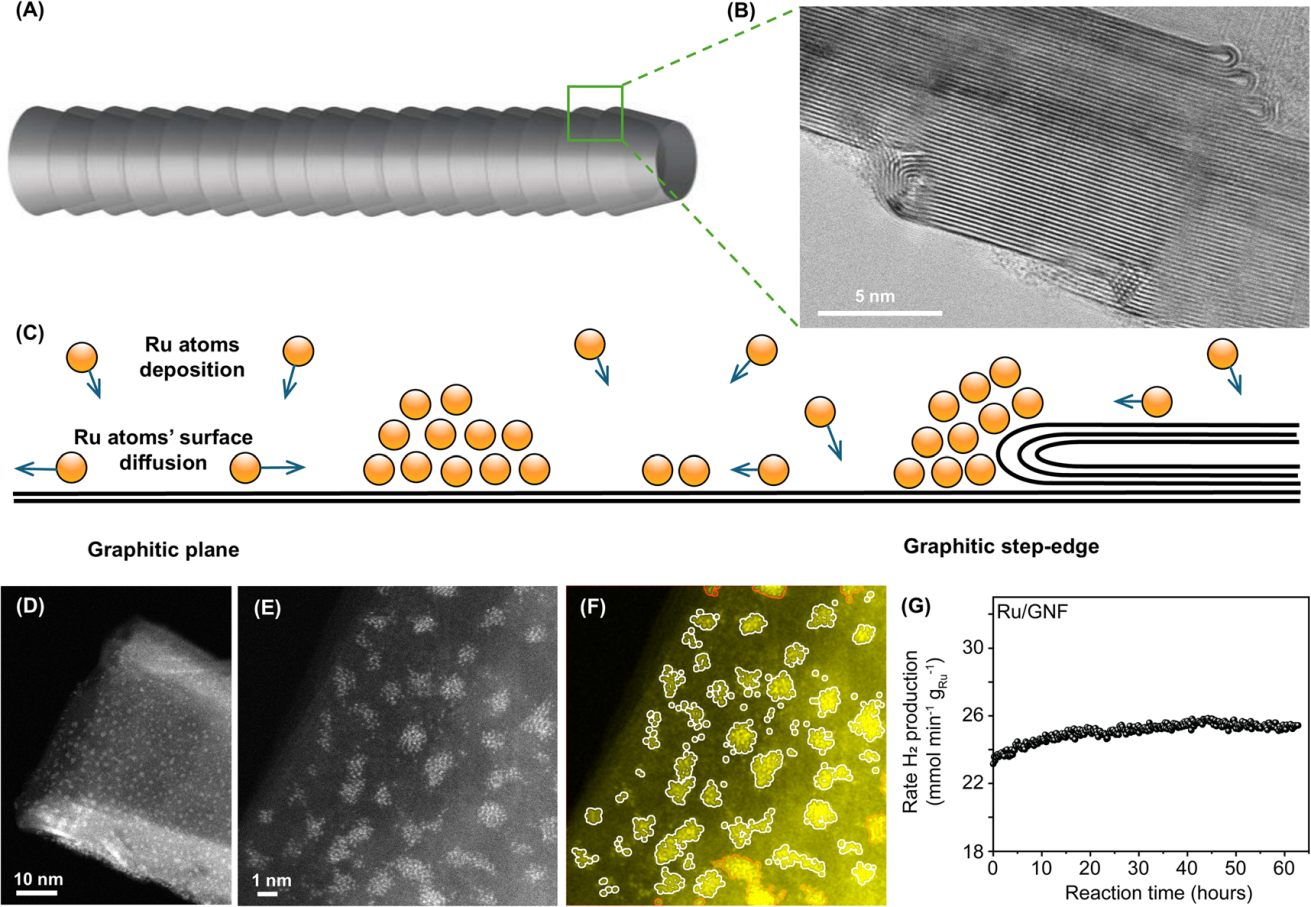
(A) Schematic and (B) TEM image show that GNF consists of a set of stacked graphitic cones. (C) Schematic representation of atomic deposition process of Ru onto a graphitic surface leading to self-assembly of nanoclusters at room temperature. (D) Annular dark field aberration-corrected STEM (AC-STEM) images of a GNF with Ru nanoclusters. GNF has a cylindrical shape with a hollow interior. High-magnification AC-STEM image of Ru nanoclusters on GNF, raw unprocessed (E) and processed by a custom Python program with Ru atoms and nanoclusters marked by white perimeters (perimeters of clusters that extend to outside the field of view are red) (F). The formation rate of H_2_ variation in the ammonia decomposition reaction catalysed by Ru/GNF plotted against reaction time (G).

Using magnetron sputtering, Ru bulk metal was dispersed to an atomic state, and Ru atoms were deposited directly onto the support material, such as graphitised carbon nanofibers (GNF) (Section S1, ESI file†). This approach allows for a solvent-free assembly of metal nanoclusters with no additional agents, such as ligands or counterions, thus yielding pure metal in direct contact with support material.^11^ GNF consists of a set of stacked graphitic cones (Fig. 1A and B) which has been shown to improve stability,^12^ selectivity,^13^ or reusability^14^ of Pt, Pd, Rh, Cu, Au, Ru, Mo, and other catalysts in thermal or electrochemical catalysis.

Inspection of as-prepared Ru/GNF indicates that during Ru deposition metal atoms diffuse on the hexagonal lattice of the support until they become immobilised at defect sites. This results in the nucleation of metal nanoclusters, the size of which is determined by the surface density of deposited metal atoms, the density of defects, temperature, and metal-supporting bonding energy, as predicted by the kinetic theory^15^ (Fig. 1C).

Prior to the catalytic testing for ammonia decomposition, catalysts were reduced under H_2_ for 1 hour at 450 °C, a commonly used protocol to activate catalyst before admitting reactants.^10,16^ The catalyst was then cooled to 50 °C, and 5% ammonia in argon was passed over the catalyst before the temperature was ramped. The catalytic activity of Ru on GNF was evaluated by measuring the production rate of H_2_ at fixed temperature of 450 °C, typical for this reaction.^17^ Interestingly, Ru/GNF shows an increase in catalytic activity over 20 hours of reaction after which it levels off and stays stable (Fig. 1G). This is unusual behaviour as compared with ruthenium on traditional catalyst supports, such as Ru/CeO_2_, which shows a progressively decreasing activity over time (Fig. S9, ESI file†). To understand the atomic mechanisms behind Ru/GNF self-improving activity, the catalytic material was examined by electron microscopy at different stages of the process.

### Identical location analysis of the catalyst

**Fig. 2:**
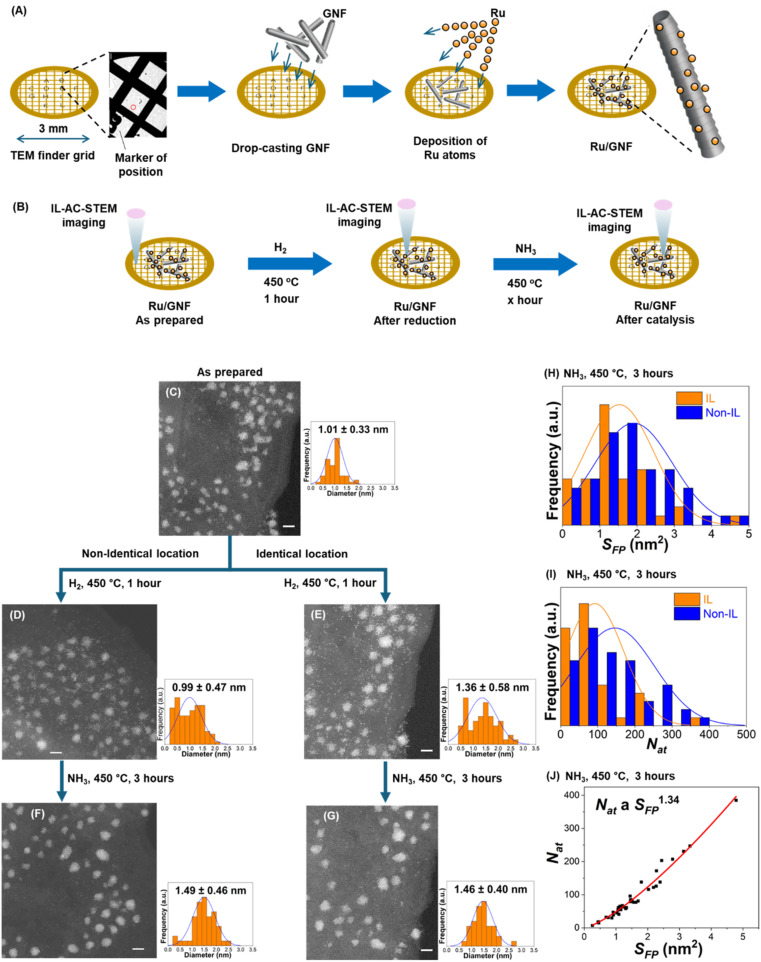
(A) Workflow of the Ru/GNF catalyst preparation on the TEM finder grid. (B) IL-STEM imaging of the nanoclusters after different stages of the reaction. AC-STEM images of as-prepared Ru/GNF (C) and after having been subjected to H_2_ at 450 °C (D and E) and subsequently NH_3_ at 450 °C (F and G) and imaged in non-identical (left) or identical (right) locations, respectively, with corresponding size distribution diagrams shown beside each STEM micrograph (scale bar, 2 nm). Distributions of the footprint area, *S*_FP_ (H) and total number of atoms, *N*_at_ (I) in Ru nanoclusters after 3 hours NH_3_ at 450 °C measured for micrographs in identical and non-identical locations. Correlation of the *N*_at_ and *S*_FP_ of Ru nanoclusters after 3 hours NH_3_ at 450 °C (J), where *R*^2^ is 0.971.

It is conventional to use electron microscopy to study changes in the size of catalyst particles. This involves comparing TEM images before and after a reaction. Electron microscopy is a powerful tool that can measure changes with atomic precision at the single particle level. However, a heterogenous catalyst always has some degree of polydispersity, *i.e.* particles are non-identical. Therefore, structural information for individual catalytic particles is typically averaged in the distribution of sizes and shapes. The alternative to the ensemble averaging analysis is to use TEM imaging in the same areas of the sample before and after each reaction stage. We prepared the Ru/GNFs catalyst directly on TEM grids (Fig. 2A and B), which allows us to average information for an ensemble of particles or study the evolution of individual particles in identical locations before and after the reaction. This provides a series of stop-frame images elucidating dynamics at the single-particle level. GNF supports, consisting of a highly conducting and chemically stable graphitic lattice, lend themselves to this approach very well due to their low STEM contrast and high electron beam stability. In addition, the positions of GNFs on the TEM finder grid with alphanumerically labelled areas allow us to return to the same set of nanoclusters before and after the reaction.^18^ Below, we show that applying the IL-STEM approach to Ru on GNF at various reaction stages can provide structural and dynamic information for individual particles while representing the overall sample (Fig. 2C–I).

Using this approach, the evolution of nanoclusters was studied in several uniquely defined areas of the sample, each approximately 20 nm by 20 nm, by imaging the same area before and after the reaction (Fig. 2C, E and G). STEM image analysis in this format allows qualitative assessment of atomic order in individual nanoclusters from the image and FFT plot, as well as quantitative analysis of the number of atoms (*N*_at_) comprising the nanocluster and its footprint (*S*_FP_) (Fig. 2H and I), and the number of atomic layers (*N*_l_) which can be deduced from these parameters based on the hexagonal close packed (hcp) lattice of ruthenium (Section S3, ESI file†). Next, the comparison of the IL and ensemble averaging (non-IL), approaches for nanocluster size analysis indicates a similar trend but differs numerically. For example, the average size of the nanoclusters in the same area of GNF increases by 35% after the H_2_ treatment step and a further 7% after the first three hours of the NH_3_ decomposition reaction (Fig. 2C, E and G), while the same analysis for different areas of GNF selected at random shows no changes after hydrogen treatment and an increase of 50% after 3 hours of reaction (Fig. 2C, D and F). Changes in nanoclusters' distribution within the same area hold a greater significance because we are observing the evolution of the specific set of atoms and nanoclusters in the same local nano-environment. Hence, the results obtained from the IL analysis should be considered more definitive for understanding the atomistic mechanisms of nanocluster evolution. Furthermore, changes in macroscopic properties of Ru/GNF, such as catalyst activity, can be linked to information obtained from local scale analysis without the need to gather statistics from many random areas.

### Individual Ru nanocluster evolution

**Fig. 3:**
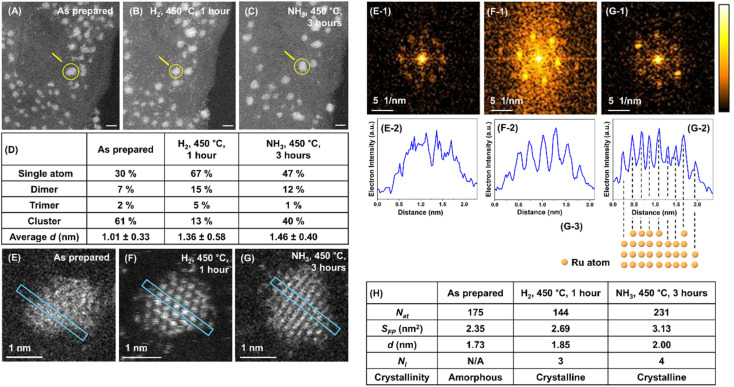
IL-STEM images of Ru/GNF at different stages: as-prepared (A), after 450 °C in H_2_ (B), and after 450 °C in NH_3_ (C) (scale bar, 2 nm). A tabulated summary of changes in the population of single atoms, dimers, trimers and nanoclusters, and the nanoclusters' average *d* for each stage (D). An example of IL-STEM analysis for the evolution of a specific single Ru nanocluster (marked with the arrow in A–C) through different reaction stages (E–G), with corresponding FFT patterns (E1–G1) and intensity line profiles (E2–G2) cut along the directions marked on STEM images. Atomic columns in nanocluster (F) are aligned parallel to the electron beam of STEM, which allows determining the number of Ru atoms in each column (F3). A summary of key structural parameters for the single Ru nanocluster at different reaction stages (H), where N/A means not applicable.

The IL-STEM approach's most important feature is its ability to track the evolution of individual nanoclusters step by step. If the nanocluster remains in approximately the same position with respect to the landscape of the GNF support (Fig. 3A–C), it can be located and examined in detail after the reduction in H_2_ and after the reaction in NH_3_ (Fig. 3E–G). The degree of metal atom ordering can be visually assessed from the STEM images or from the FFT of the images (Fig. 3E1–G1).^19^ In addition, in cases where atomic columns in the metal nanocluster align with the direction of the electron beam, the intensity line profile drawn across the nanocluster can be used to count the number of atoms in each atomic column from its peak intensity (Fig. 3G3).^20^ Simultaneously, the total *N*_at_ and area of *S*_FP_ can be conveniently determined for the same nanocluster directly from the integral intensity of the STEM image and perimeter measurement, providing a full description of its structure (Fig. 3H).

**Fig. 4:**
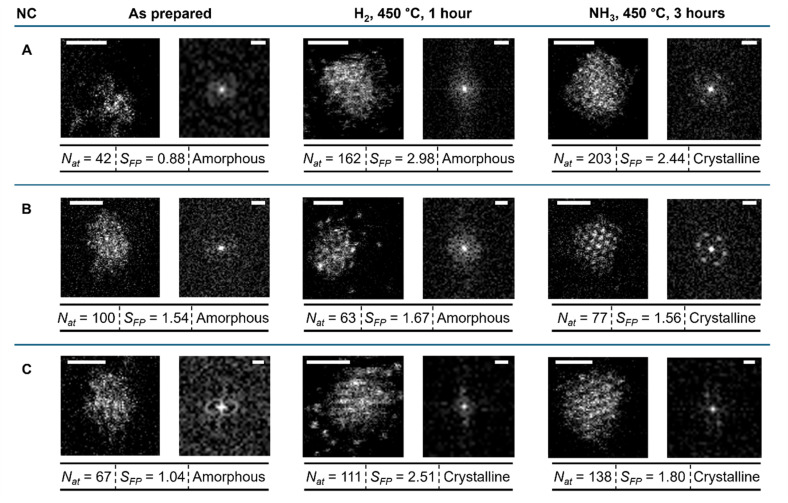
Evolution of Ru/GNF after H_2_ treatment for 1 hour and NH_3_ decomposition reaction for 3 hours: identical location observation of individual nanocluster. STEM images with FFT patterns shown on the right side, and key nanocluster parameters below each image. The scale bar in the STEM image is 1 nm. The scale bar in FFT patterns is 5 nm^−1^.

For example, changes in *S*_FP_ and *N*_at_ can be directly traced for 9 individual nanoclusters (Fig. 4 and S3†), showing various types of behaviour. For *N*_at_, some undergo an increase and some decrease in H_2_, followed by an increase in NH_3_, while for *S*_FP_, the majority undergoes an increase in H_2_, followed by a decrease in NH_3_. This implies that during the stage of NH_3_ decomposition reaction, the nanocluster becomes more compact (increasing *N*_at_ with decreasing *S*_FP_).

**Fig. 5:**
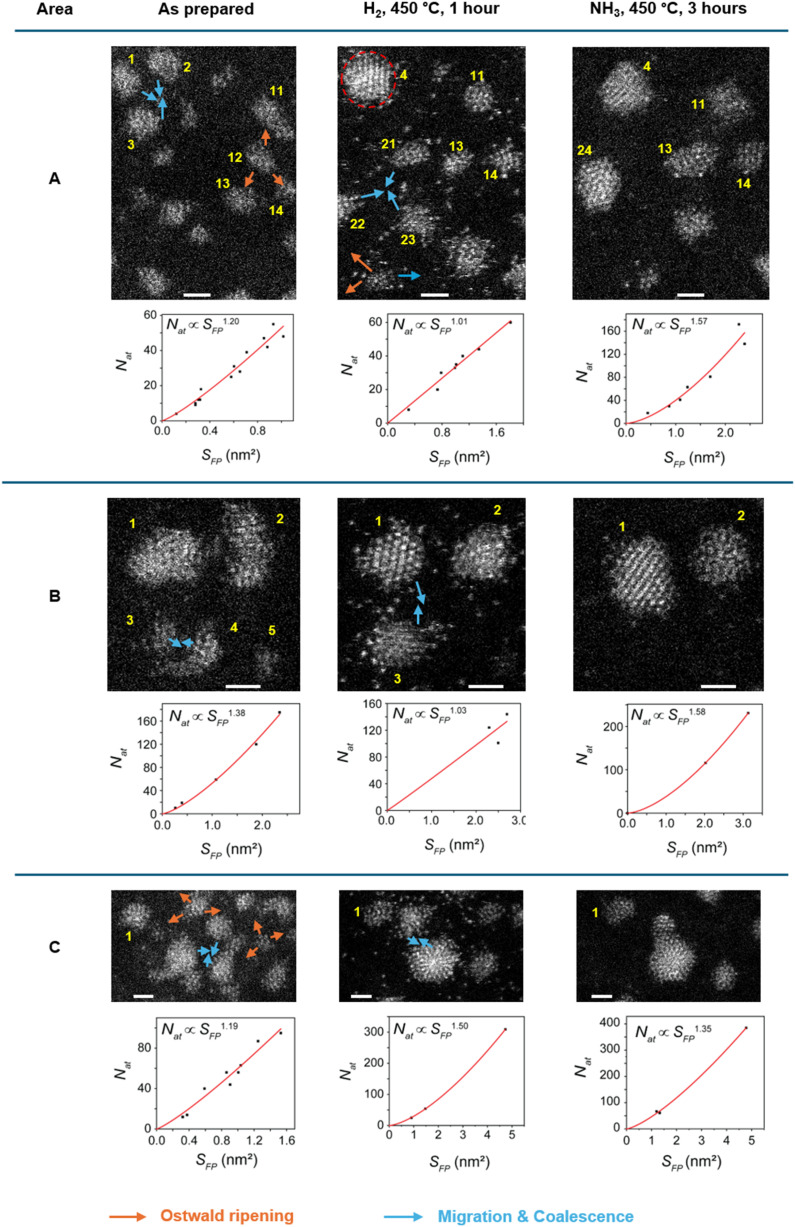
Evolution of groups of nanoclusters after H_2_ treatment for 1 hour (middle column) and NH_3_ decomposition reaction for 3 hours (right column): identical location observation. Changes in nanoclusters are indicated by arrows (orange = Ostwald ripening; blue = migration and coalescence). A plot correlating *N*_at_ and *S*_FP_ is shown for each area beneath the micrographs. Scale bar is 1 nm. The clusters circled in red were not included in the analysis because it extends beyond the field of view.

By plotting *N*_at_ against *S*_FP_ (Fig. 2J and 5), one can deduce information about the 3D shape of the nanoclusters. The plot can be fitted with a power law with an exponent of 1.3, suggesting that the nanoclusters are not cylindrical or disk-shaped with *S*_FP_-independent height but rather closer to hemispherical or pyramidal. A deeper level of analysis can be achieved from the image intensity profile, allowing for the intensity of the atomic columns of Ru to be examined. This intensity is proportional to the number of atoms in the respective column, thus providing the 3D shape of the nanocluster as a pyramid with stepped sides and a flattened top, with the *N*_l_ increasing from 1 at the base to 4 at the apex (Fig. 3G3). Analysis of other examples of well-defined, trackable nanoclusters reveals that atomic transformations are strongly dependent on the local environment, with many nanoclusters following the same general trend as described above, *i.e.* both crystallinity (albeit just for a few nanoclusters) and *S*_FP_ increasing after H_2_ treatment, with a further increase of crystallinity but a decrease of *S*_FP_ after ammonia reaction (Fig. 4 and S3†).

Furthermore, analysing the AC-STEM Ru images allows us to evaluate the degree of atomic order in the nanoclusters. For example, a typical Ru nanocluster formed at room temperature on the carbon support lacks atomic ordering (Fig. 3E). However, after the treatment in H_2_, ordered columns of atoms emerge (Fig. 3F). FFT image analysis confirmed that the overall trend is that the nanoclusters' degree of crystallinity increases, especially after the NH_3_ step of the reaction (Fig. S3 and 5†).

### Evolution of Ru nanocluster in groups

IL-STEM determined that the decrease of nanocluster *S*_FP_ and the increase of *N*_at_ and *N*_l_ contribute to the nanocluster's increasing crystallinity, as Ru nanoclusters become more compact. However, it does not explain the source of the extra Ru atoms. This was answered by considering the nearest neighbourhood of each nanocluster. We split the overall area under investigation into 9 distinct sub-areas, such that changes for several nanoclusters can be tracked from one step to another. Overall, we identified three types of behaviour of nanoclusters: (1) migration followed by coalescence, (2) migration without coalescence, and (3) Ostwald ripening. The latter appears more prevalent during the H_2_ reduction step as a particular nanocluster disperses into atoms feeding into nanoclusters nearby (Fig. 5). Based on changes in the size of nearest neighbours, the disappearing nanocluster typically transfers its atoms to at least three adjacent nanoclusters. Migration and coalescence events both appear to be present during the H_2_ reduction and NH_3_ decomposition and typically involve two or three nanoclusters merging into one (blue arrows in Fig. 5). Identical location analysis revealed that during this mechanism, two or three nanoclusters move towards each other, and these become merged into a single structure.

Both Ostwald ripening and migration followed by coalescence decrease the number of nanoclusters while increase the *N*_at_ per cluster and height. Thus, inter-cluster separations become larger, with a wider expanse of carbon support opening up between the nanoclusters. Inspection of the space created by disappearing nanoclusters reveals the presence of single Ru atoms adsorbed on carbon support after H_2_ conditions. In contrast, the fraction of single atoms after NH_3_ decomposition reaction is significantly lower (Fig. 5).

### Ru nanocluster evolution over 12 hours of NH_3_ decomposition reaction

**Fig. 6:**
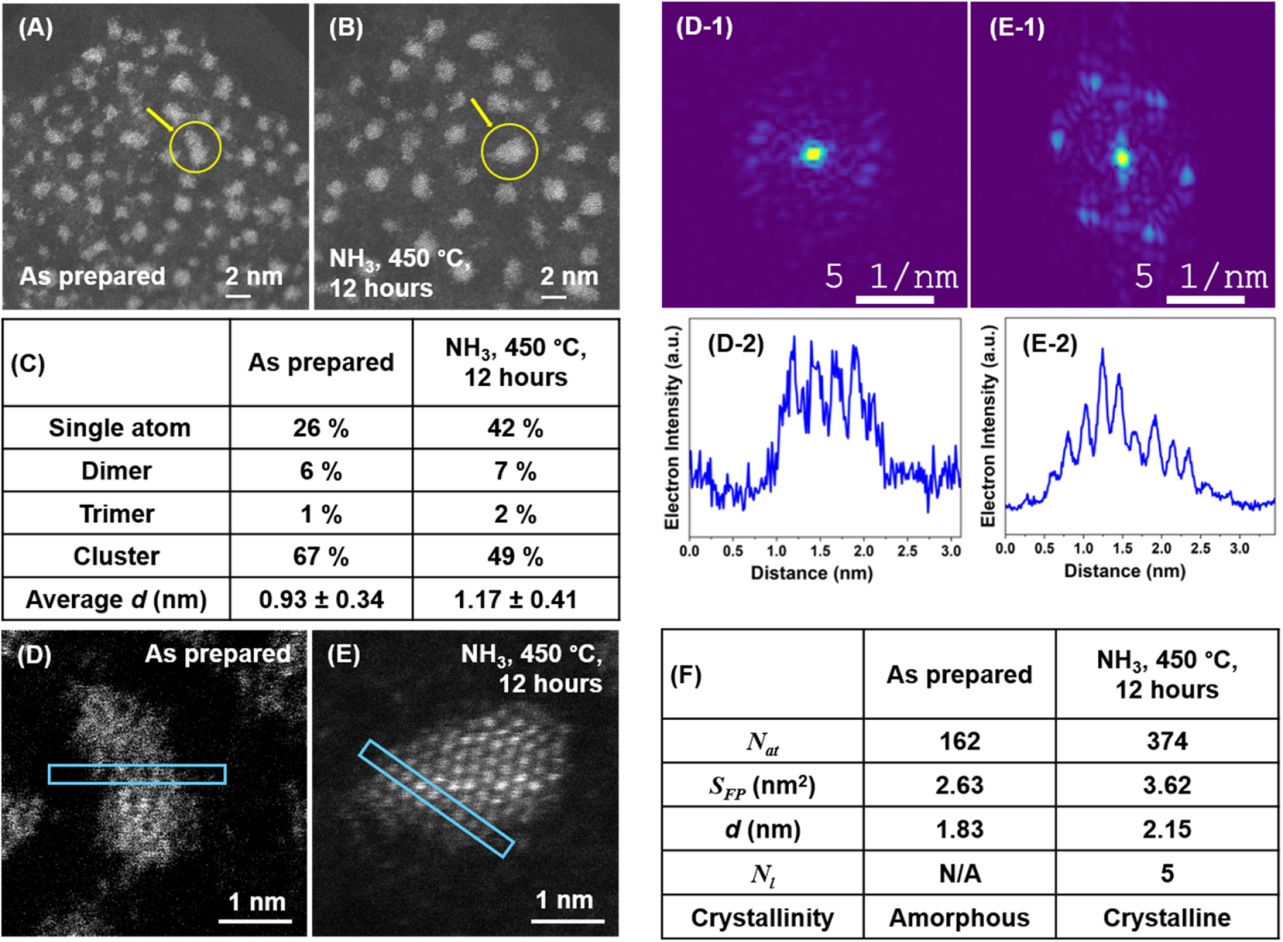
IL-STEM images of Ru/GNF at different stages: as-prepared (A) and after 450 °C in NH_3_ (B). A tabulated summary of changes in the population of single atoms, dimers, trimers and nanoclusters, and the nanoclusters' average *d* for each stage (C). An example of IL-STEM analysis for the evolution of a specific single Ru nanocluster (marked with the arrow in A and B) through different reaction stages (D and E), with corresponding FFT patterns and intensity line profiles (D2 and E2) cut along the directions marked on STEM images. A summary of key structural parameters for the single Ru nanocluster at different reaction stages (F), where N/A means not applicable.

We performed identical location measurements for Ru/GNF as-prepared and after 12 hours of the ammonia decomposition reaction (Fig. 6). Remarkably, the nanocluster *S*_FP_ does not increase beyond that of 3 hours reaction (Fig. 6C). The pyramidal shape with well-defined edges due to atomic ordering with steps appears very stable under the ammonia decomposition reaction conditions (Fig. 6E).

## Discussion

Our study indicates that the catalytic activity of ruthenium nanoclusters on carbon increases with time, which contrasts with Ru on metal oxides, such as Ru/CeO_2_ whose activity gradually decays – a typical behaviour of heterogenous catalysts (Fig. S9†). Understanding the source of the increasing activity is crucial, as it can provide a blueprint for designing new types of catalysts. Our electron microscopy measurements showed that the average number of atoms per Ru nanocluster increases in the initial 3 hours of the reaction (Fig. 2C–I and 3D) and does not increase substantially beyond this over 12 hours of reaction (Fig. 6C). Hence, the fraction of surface Ru atoms per nanocluster decreases and levels off, contradicting the observed rise of the activity of Ru/GNF. To explain this unexpected phenomenon, a simple averaging of structural information does not prove to be fruitful. Indeed, the ensemble-averaging analysis masks essential features of the nanocatalysts, making it challenging to relate nanoscale structure to the macroscopic properties of the material, including catalytic activity. For instance, particle average size or diameter concepts cannot be fully described at the nanoscale because of the non-spherical, irregular shapes of the metal nanoclusters, and it does not carry information about the third dimension (particle height). Furthermore, the process of averaging obscures details about various local particle environments, such as the proximity and number of nearby particles. Since the local environment can significantly impact the behaviour of individual particles, it is challenging to discern the atomic mechanisms responsible for these changes through ensemble averaging analysis.

**Fig. 7:**
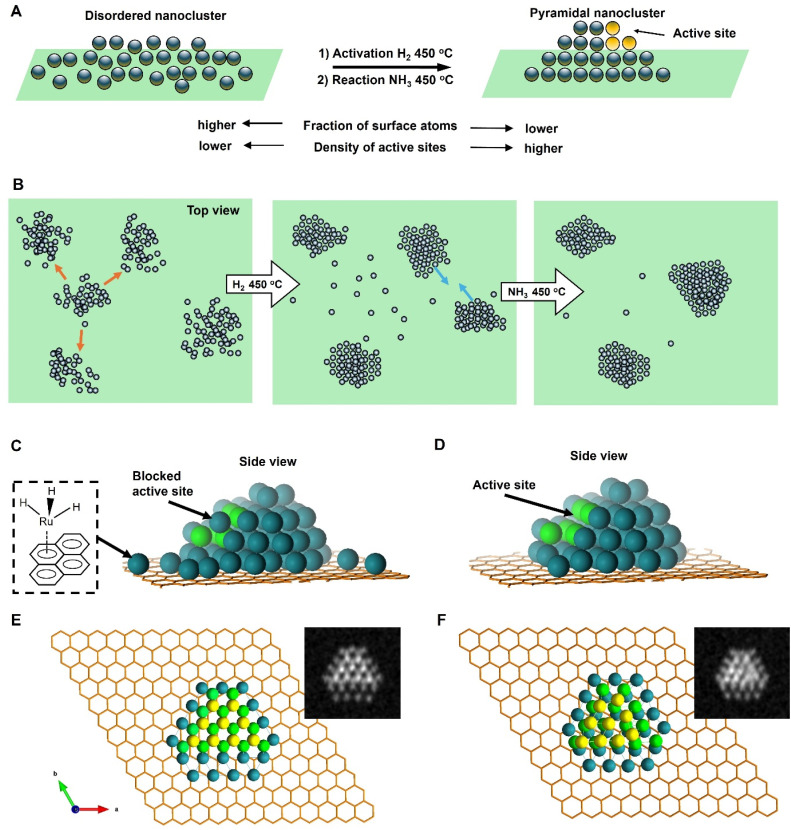
A schematic diagram illustrating changes in Ru nanoclusters in the activation step (H_2_, 450 °C) and during the reaction (NH_3_, 450 °C) (A). The nanocluster becomes more compact with the fraction of surface atoms decreasing and a truncated pyramidal shape clad with atomic steps evolving. Ostwald ripening (orange arrows) and coalescence of nanoclusters (blue arrows) are responsible for the pyramidalisation process, with the population of single Ru atoms increasing in the activation step followed by a decrease during the reaction (B). The state of the Ru nanocluster after activation in H_2_ (C). Most atoms are arranged in the lattice of a truncated pyramid, but there is a large number of single Ru atoms stabilised by hydride ligands and chemisorbed on the carbon surface (inset) and the surface of the pyramid, which blocks some active sites (atoms highlighted light green). During the reaction in ammonia, single Ru atoms become integrated into atomic lattices of nanoclusters, with the edges and facets of the pyramids becoming more sharply defined, increasing the density of active sites (D). A structure of Ru_50_ nanocluster adsorbed on graphene before (E) and after (F) relaxation calculated by DFT. Ru atoms in the bottom layer (dark green) bind strongly to the carbon lattice while maintaining a distorted hexagonal arrangement, but Ru atoms in the second (light green) and third layers (yellow) adjust their positions more substantially during the relaxation to maximise metal–metal bonding. STEM images simulated from the DFT models are shown in the insets.

Fortunately, IL-STEM imaging provides a solution to the issue of ensemble averaging by enabling the tracking of individual catalyst particles' evolution from one stage of the reaction to another (Fig. 2C, E and G). In addition, considering that the diameter of the TEM grid is macroscopic, *ca.* 3 mm across, the IL-STEM analysis can be performed on several areas of the grid. Hence such measurements are completely independent of each other (Fig. 2C–I) which ensures the experimental reproducibility as well as representativeness of the area chosen for deep analysis of the whole macroscopic sample. Using this approach allowed us to assess the evolution of individual Ru nanoclusters during catalyst activation (450 °C, H_2_) and the initial phase of the ammonia decomposition reaction (450 °C, NH_3_). We employed nanoclusters' footprint *S*_FP_ alongside the total number of atoms in the nanocluster, *N*_at_ that can be estimated from the integral intensity of STEM image, as more meaningful descriptors instead of the average size or diameter. Considering a close-pack of Ru metal atoms in the base layer of nanocluster, the number of layers *N*_l_ can be inferred from *S*_FP_ and *N*_at_ (ESI, Section S3†). These parameters, combined with the line intensity profiles, provide a comprehensive description of the three-dimensional shape of the nanoclusters and their evolution in the reaction, which was monitored by IL-STEM approach (Fig. 3E2–G2, 6D2 and 6E2). This method led us to conclude that the *N*_at_ in Ru nanoclusters increases due to the expanding *S*_FP_ and the increasing *N*_l_ during H_2_ treatment. However, during the NH_3_ decomposition reaction, *S*_FP_ decreases while *N*_at_ continues to grow so that the nanoclusters become taller and progressively pyramidal, with stepped edges (Fig. 7A). The correlation of *S*_FP_ with *N*_at_ showed an increase of the scaling power *α* from 1.20 to 1.57, 1.38 to 1.58 and 1.19 to 1.35 in *N*_at_ ∼ (*S*_FP_)^*α*^ (Fig. 5), which also confirms that clusters become more three-dimensional.

The determination of the precise atomic structure of the nanoclusters, such as in Ru/GNF, is challenging. Our IL-STEM imaging and FFT image analysis clearly indicate an increasing crystallinity of the nanoclusters during the reaction, but their structural analysis is complex. However, when the atomic columns of Ru in nanoclusters are parallel to the electron beam (Fig. 3F and G), the observed STEM images were consistent with Ru hcp lattice structure (Fig. S8†).

It is also important to consider the relationship of Ru atoms with the carbon lattice of GNF. Previous studies on bulk Ru have demonstrated that graphene layers grow epitaxially on the Ru (0001) surface.^21^ Our DFT calculations, performed for Ru_50_ nanoclusters on graphene, clearly demonstrate that Ru atoms in the bottom layer bond firmly to the carbon atoms (dark green), as seen in structural changes before and after relaxation (Fig. 7E and F). This suggests that the graphitic lattice of GNF can, in principle, influence the symmetry and interatomic distances in the base layer of Ru island. Importantly, the second and third layers of atoms in Ru_50_ deviate significantly from the structure of the bottom layer (light green and yellow, respectively; Fig. 7F). While retaining the general structural features of the bulk Ru, the sub-2 nm nanoclusters appear to be significantly plastic due to a high fraction of surface atoms and strong bonding with carbon causing displacement of Ru atoms from hcp lattice positions both in the lateral and vertical directions. This explains the greater disorder in smaller nanoclusters observed in AC-STEM images.

Our IL-STEM approach enables us to investigate the atomic mechanisms of nanocluster pyramidalisation by considering the evolution of each nanocluster within the context of its nearest neighbours (Fig. 5). As the reactions occur at 450 °C, Ru atom exchange between nanoclusters and direct cluster–cluster interactions are both likely to occur. The Ru bonding energy with the carbon support can be estimated by that of Ru-graphene bonding of 189 kJ mol^−1^,^22^ but it is expected to be much higher at places of defects, such as a mono-vacancy (835 kJ mol^−1^)^22^ or step-edges of GNF. Therefore, the nanoscale landscape of the support would inevitably lead to the re-distribution of metal atoms on the surface once the temperature exceeds their surface diffusion barrier (Fig. 7B). Each area in IL-STEM can be split into several sub-areas where the structural evolution of a Ru nanocluster can be considered in conjunction with the evolution of its neighbours as they affect each other. For example, the area shown in Fig. 5a exhibits trackable nanoclusters. During the catalyst activation in H_2_, three nanoclusters 1, 2 and 3 in the top-left corner moved and coalesced into a single nanocluster 4, and a nanocluster 12 on the right side disintegrated with three of its nearest neighbours, 11, 13 and 14, gaining atoms, such that the total number of nanoclusters in this area has reduced. An important feature of heating in H_2_ is the explosion of the population of single atoms occupying the inter-cluster spaces (Fig. 5, middle column). This correlates with the previous environmental STEM study of Ru nanoparticles on graphitic and amorphous carbon that revealed a surprisingly large fraction of single atoms at 450 °C in an H_2_ : N_2_ 3 : 1 gas mixture at 1–20 Pa.^23^ Returning to the same area after 3 hours of NH_3_ decomposition catalysis reveals that 3 nanoclusters, 21, 22 and 23 have coalesced into a single clusters cluster 24. The number of single Ru atoms drastically decreased, and Ru nanoclusters became significantly more faceted, with sharp edges. The pattern of these transformations repeats from area to area, with the frequency of coalescence events seems to become more prevalent than Ostwald ripening under the reaction conditions (Fig. 5).

The single atoms emerging under H_2_ are likely produced due to the ruthenium hydride complex bonding strongly to the carbon support (Fig. 7C), as Ru atoms stabilised with hydride ligands is known to form π-bonds effectively to aromatic molecules,^24^ which in our case is served by the graphitic lattice of the GNF support. Under NH_3_, the hydride complex breaks down, with most of the single Ru atoms returning to the nanoclusters (Fig. 7D), thus boosting their size and crystallinity, as evident from IL-STEM images. The fact that the growth of nanoclusters does not progress significantly beyond 4 nm^2^*S*_FP_ even after 12 hours of the reaction indicates a significant stabilising effect of the GNF surface, which limits the surface diffusion of metal atoms, hence leading to an equilibrium state with a narrow size distribution of metal nanoclusters.^15^

The role of the edges of the Ru hcp planes, particularly in a step-like arrangement, is extremely important in ammonia synthesis and decomposition reactions.^25,26^ The strong binding energy of atomic nitrogen on Ru means that the rate-determining step in the ammonia decomposition reaction is the recombination of N atoms to N_2_,^27^ which takes place on so-called step active sites with favourable electronic and geometric properties for desorption.^28^ The evolution of Ru on hexagonal boron nitride (hBN) driven by the epitaxial relationship between Ru hcp and hexagonal hBN lattices has recently been reported to cause the formation of ∼10 nm hexagonal bifrustum nanoparticles, with long sharp edges providing a higher rate of hydrogen production from ammonia than more rounded nanoparticles.^4^ In our case, the size of Ru nanoclusters is significantly smaller, in the region of 1.0–1.3 nm at the start of the process, such that a high fraction of Ru surface atoms is obtained in our nanoclusters. Importantly, our IL-STEM measurements allow monitoring the evolution of individual nanoclusters with atomic resolution, quantifying changes in their structure during catalyst activation and early stages of the reaction. This reveals the formation of truncated nano-pyramids clad with a series of atomic steps on every side (Fig. 7A). In light of IL-STEM analysis, both for individual nanocluster dynamics in isolation or coupled with the immediate neighbourhood, the answer to the question of what changes in Ru nanoclusters are responsible for the catalytic activity increase during the reaction's early stages becomes clearer. The overall trend is that the *N*_at_ and *N*_l_ of the nanoclusters increase while the *S*_FP_ decreases, which is correlated with the increase of the atomic order in the nanoclusters. As this process decreases the fraction of surface atoms, it should be expected to lead to lower catalytic activity. However, the atomic ordering process allows crystal planes to be developed in nanoclusters, which are arranged in a stepped structure. The stepped structure, developed from flattened disordered Ru nanoclusters during the reaction, is clearly visible in single-particle IL-STEM imaging and line profile analysis (Fig. 3G2, 6E2 and S5†). As each step on the Ru nanocluster represents a potential active site, where the reaction proceeds several times faster than on the flat crystal plane or a disordered metal surface. This compensates for the decrease of the fraction of surface atoms, thus explaining the increasing activity of Ru/GNF catalyst during the reaction. Our DFT modelling revealed that the Ru nanoclusters on carbon exhibit significant plasticity (Fig. 7E and F). As a result, the exact atomic configuration in the active site of Ru/GNF may differ from the idealised models used for larger nanoparticles, which are based on bulk hcp Ru. Subtle sub-Angstrom displacements of Ru atoms within the nanocluster are difficult to discern experimentally, but they may have significant implications for the kinetics of catalysis.

We believe that the single Ru atoms on GNF play no role in catalysts, as their population drastically decreases as the catalyst becomes more active (Fig. 3D). Moreover, Ru atoms of the surface of nanocluster which aren't in the lattice may be responsible for blocking Ru active sites at the early stage of the reaction (Fig. 7C), and therefore, as these atoms become incorporated in the crystal lattice of nanoclusters, accessibility of active sites improves, boosting the ammonia decomposition rate. The gradual evolution of the nanocluster's *S*_FP_ stabilising at around 2–4 nm^2^ (*ca.* 1.6–2.3 nm in *d*) maximises the number of active sites per mass of Ru metal in the system, as an optimum size for this was predicted to be around 3 nm.^29^ The stepped structures persist over a long time, as shown by IL-STEM analysis for 12 hours of ammonia decomposition reaction (Fig. 6). This means that the shapes that evolved during the catalyst activation in the early stages of the reaction are stable on the surface of graphitised carbon under the reaction conditions. This helps to explain the self-improving activity of Ru/GNF observed in our reaction kinetics measurements.

## Conclusion

The high volumetric energy density of ammonia, compared to hydrogen and other zero-carbon technologies such as lithium batteries, gives it the potential to establish a new energy economy in the near future.^6,30^ Ruthenium catalysis offers energy-efficient methods to break down ammonia into its elements, H_2_ and N_2_, on-demand, at both small and large scales. In this work, we demonstrated that ruthenium atoms deposited directly onto the graphitic surface of GNF self-assemble into clusters with a *S*_FP_ of about 1 nm^2^ and an irregular shape. The Ru/GNF material has shown high catalytic activity for the ammonia decomposition reaction, exceeding the activity of Ru on metal oxide supports under the same conditions, including Ru/CeO_2_ regarded as one of the best catalysts. While Ru/metal oxide catalyst performance declined during the reaction, we demonstrate that the Ru/GNF catalyst increases its activity over 20 hours of the ammonia decomposition reaction. As the traditional electron microscopy analysis methods cannot explain this phenomenon, we employed the identical location aberration-corrected STEM imaging to follow the evolution of Ru nanoclusters through different stages of the reaction process to elucidate the origin of the increasing activity. Our data show that activation of the as-prepared Ru nanoclusters on GNFs at 450 °C in H_2_ induces the ordering of Ru atoms within nanoclusters as well as the formation of a large fraction of single Ru atoms scattered across the graphitic support. The latter is unlikely to play any significant role in the increased catalytic activity of Ru/GNF, as they are only present in the very initial part of the reaction. Investigation of the evolution of individual, well-defined nanoclusters of Ru revealed that during the reaction the *N*_at_ and *N*_l_ mainly increased, thus reducing the fraction of surface Ru atoms. The quantitative AC-STEM image analysis in identical locations demonstrated the atomic ordering in the edges of the nanoclusters and the development of stepped structure, leading to the increased density of active sites, which more than compensates for the loss of surface area and boosts the catalytic activity of Ru/GNF measured. Furthermore, identical location AC-STEM analysis of groups of nanoclusters within the area of the nearest neighbourhood revealed that during the catalyst activation in H_2_ both Ostwald ripening and coalescence are in action. However, the coalescence of nanoclusters is becoming the dominant underlying mechanism for the catalyst stepped structure formation under the reaction conditions, leading to its self-improved activity. Importantly, we demonstrated that GNFs stabilise the Ru stepped structure, not allowing them to grow beyond *c.a.* 4 nm^2^*S*_FP_. This mechanism plays a crucial role in the enhanced activity and extended stability of the catalyst, opening a path for designing highly active and durable catalysts for ammonia decomposition reactions.

## Data availability

Additional data can be obtained from the authors upon request.

## Author contributions

Yifan Chen: investigation, data curation, methodology, visualisation, writing the original draft. Benjamin J. Young: investigation, data curation, methodology, writing the original draft. Gazi N. Aliev: investigation, data curation, methodology, visualisation, manuscript review & editing. Apostolos Kordatos: formal analysis, methodology, manuscript review & editing. Ilya Popov: investigation, formal analysis, data curation, methodology, validation, manuscript review & editing. Sadegh Ghaderzadeh: formal analysis, methodology, manuscript review & editing. Thomas Liddy: investigation, data curation. William J. Cull: formal analysis, visualisation, manuscript review & editing. Emerson C. Kohlrausch: investigation, methodology, manuscript review & editing. Andreas Weilhard: methodology, manuscript review & editing. Graham J. Hutchings: methodology, manuscript review & editing. Elena Besley: methodology, formal analysis, validation, supervision, manuscript review & editing. Wolfgang Theis: investigation, data curation, formal analysis, methodology, software, supervision, validation, visualisation, manuscript review & editing. Jesum Alves Fernandes: conceptualisation, investigation, supervision, validation, writing the original manuscript, manuscript review & editing. Andrei N. Khlobystov: conceptualisation, investigation, supervision, writing the original manuscript, manuscript review & editing.

## Conflicts of interest

There are no conflicts to declare.

## Supplementary Material

SC-016-D4SC06382A-s001
