# Supplementary material for: Evolution of amorphous ruthenium nanoclusters into stepped truncated nano-pyramids on graphitic surfaces boosts hydrogen production from ammonia
Source: Chem Sci. 2025 Jan 9;16(6):2648–60. doi: 10.1039/d4sc06382a (PMC11718984; doi:10.1039/d4sc06382a)
Supplement: SC-016-D4SC06382A-s001 [file SC-016-D4SC06382A-s001.pdf]

Supplementary Information (SI) for Chemical Science.
This journal is © The Royal Society of Chemistry 2025

Supporting Information

Evolution of amorphous ruthenium nanoclusters into stepped truncated nano-pyramids on graphitic surfaces boosts hydrogen production from ammonia

Yifan Chen[1], Benjamin J. Young[1], Gazi N. Aliev[2], Apostolos Kordatos[1], Ilya Popov[1], Sadegh Ghaderzadeh[1], Thomas J. Liddy[1], William J. Cull[1], Emerson C. Kohlrausch[1], Andreas Weilhard[1], Graham J. Hutchings[3], Elena Besley,[1] Wolfgang Theis[2]*, Jesum Alves Fernandes[1]* and Andrei N. Khlobystov[1]*

[1]School of Chemistry, University of Nottingham, University Park, NG7 2RD, UK

[2]Nanoscale Physics Research Laboratory, School of Physics and Astronomy, University of Birmingham, Edgbaston, B15 2TT, UK

[3]Max Planck Centre on the Fundamentals of Heterogeneous Catalysis FUNCAT, Cardiff Catalysis Institute, Translational Research Hub, Cardiff University, Cardiff, CF24 4HQ, UK

S1. Materials and Methods

Sample Preparation

Magnetron Sputtering was conducted using a custom-built AJA sputtering chamber equipped with a ruthenium target. The work current was maintained at 160 mA, work distance at 90 mm, and the applied power and deposition time were 2.5 s. These conditions were varied accordingly per experiment. GNFs were drop cast on holey carbon-coated Au grids (Agar scientific, H7 finder grids) for electron microscopy analysis and a thin GNF layer was drop cast on a petri dish for the catalytic experiments. In both case, we have used the same deposition parameters as described above.

Ammonia decomposition reaction catalytic test

The catalytic activity measurements for NH_3 decomposition were conducted in a quartz tube packed bed reactor (Hiden Analytical CATLAB Micro reactor) operating under atmospheric pressure. The flow rates of the gases were controlled by integrated mass flow controllers. The experiment was carried out at 723 K with weight hourly space velocity (WHSV) of 30,000 mL min^{-1} g_{cat}^{-1}. Prior to reaction tests, the catalysts were reduced in-situ at 723 K (1 K/min ramp) under H_2 flow (25 mL/min) for 1 hour, before being cooled to 323 K whilst purging with He

gas (25 mL/min) and held for 30 minutes. Finally, the gas flow was switched to an ammonia/argon mixture (5% NH_3/95% Ar) (25 mL/min), before being ramped to reaction temperature (1 K/min). The effluent gases were analysed by a mass spectrometer (Hiden Instruments QGA Quantitative Gas Analyser). The detector was calibrated for the gases (NH_3, N_2, and H_2) over a wide dynamic range spanning the analysis region.

Characterisation

Samples are imaged by a JEM-2100F TEM (JEOL, Japan) operated at 200 kV, which is equipped with a spherical aberration (Cs) probe corrector for STEM (CEOS, Germany). The probe convergence angle is 19 mrad and the collection angle range of the annular dark field (ADF) detector is 31 to 82 mrad. Aberration-corrected STEM (AC-STEM) images are captured with a scanning area of 1024 × 1024 pixel. The bright field (BF) detector is also used in parallel.

S2. AC-STEM image analysis and high-resolution TEM image characterization

A custom python program is used for quantitative analysis of the number of atoms, N_{at}, comprising the nanocluster and its footprint (Figure S1 a-b). N_{at} for each cluster was calculated by dividing the nanocluster's integrated background-subtracted image intensity by the average integrated background-subtracted image of the identified single Ru atoms. Furthermore, under our reaction conditions, we did not observe significant changes in the structure of the GNF support material, such as the opening of layers of graphene folded at the step edges, which may occur in some other reactions (Figure S1 c-f).

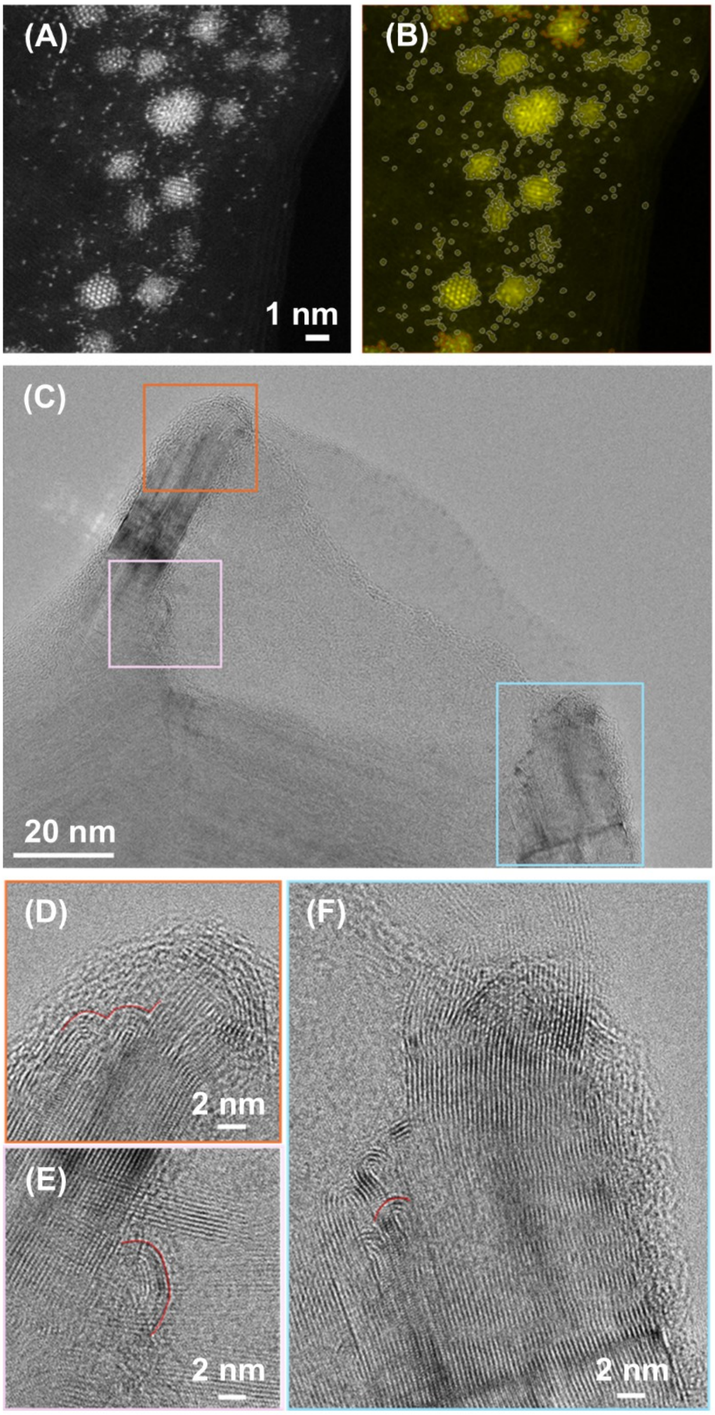

Figure S1: An example of ADF-STEM electron microscopy image of Ru/GNF used to calculate the SA:Di:Tri:NC ratio. (b) Identification of single atoms, dimers, trimers, and larger nanoclusters. (c) High-resolution TEM image with selected areas (d-f) showing graphitic step-edges in Ru/GNF after the reaction.

S3. Estimation of the number of layers in Ru nanoclusters

The number of layers, N_l, can be deduced from the total number of atoms in a cluster (N_{at}) and footprint S_{FP}. To do that we have to make certain assumptions about crystal geometry of our clusters allowing to build a structural model:

1. For the purpose of the calculations, we assumed that the base of the cluster corresponds to (001) plane of the hcp lattice.

2. We then assumed that all layers in the cluster have the same number of atoms. This allows to estimate N_l as N_{at}/N_m, where N_m is number of Ru atom in the base layer which can be calculated using equations 1 and 2.

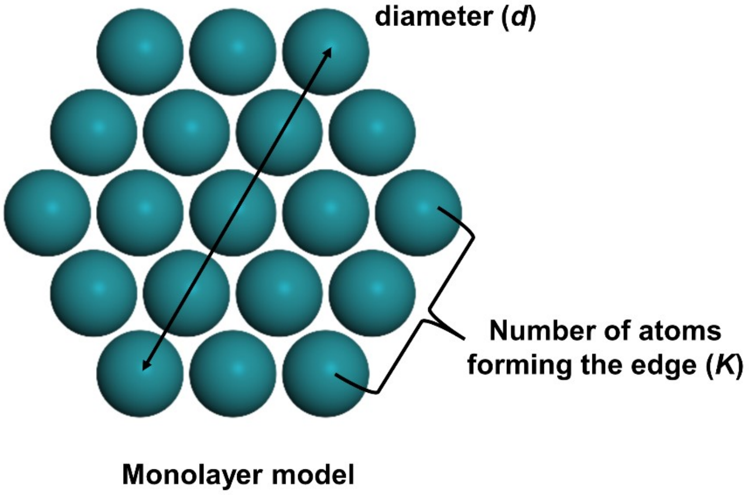

Figure S2: Monolayer Ru hcp model illustrating the determination of N_m.

$$K = \left\lfloor \frac{2\sqrt{\frac{S_{FP}}{\pi}}}{2d_{Ru-Ru}} \right\rfloor + 1 \quad (1)$$

$$N_m = 3K(K-1) + 1 \quad (2)$$

S4. Shape of Ru nanoclusters

An approximate shape characteristic for a series of nanoclusters can be deduced from the dependence of number of atoms in clusters (N_{at}) on their footprint area (S_{FP}). The number of atoms in a 3D cluster is proportional to its volume (V) if the density of Ru atoms remains the same across the considered series of clusters. For the ideal hemisphere $V \propto d^3$ and $S_{FP} \propto d^2$, where d – is diameter of the hemisphere. It means that $N_{at} \propto V \propto S_{FP}$^ (3/2). For a cylinder $V \propto h \bullet d^2$ and $S_{FP} \propto d^2$, where d is the diameter of the cylinder and h *is* its height. If the height is constant for a selected set of clusters, we get the following relationship $N_{at} \propto V \propto S_{FP}$.

By plotting experimental dependence N_{at} (S_{FP}) and fitting it with a function $N_{at} = C{*}S^{\alpha}_{FP}$ one can determine the value of α corresponding to the given set of clusters. If this value is close to 3/2 then the nanocluster height scales with its S_{FP} diameter, if it is close to 1then it is constant height.

Table S1. The R^2 of correlation of the N_{at} and S_{FP} of Ru nanoclusters in figure 5.

NC set	As prepared	H_2, 450 °C, 1 hour	NH_3, 450 °C, 3 hours
A	0.95	0.97	0.93
B	1	N/A	1
C	0.95	1	1

S5 Ru nanocluster lattice structure

To elucidate the lattice structure of the Ru nanoclusters AC-STEM images were recorded of selected nanoclusters aligned along zone-axes utilizing a double-tilt holder. Data was taken as 0 and 90 degree scan rotation pairs to allow non-linear drift distortion correction using the python implementation[1] for the approach developed by Ophus et al[2]. The procedure was applied to a Au(001) nanofilm sample to achieve a calibration of the scan coils. This calibration was applied as an affine transform to the drift-corrected image. The resulting images (Figure

S8A1-6) and their FFTs (Figure S8B1-6) were compared to simulated images (Figure S8D1-6) and respective FFTs (Figure S8E1-6) derived from spherical nanoclusters of Ru hcp bulk structure. The simulated nanoclusters were rotated to yield the best orientational match, after which their atoms' 3D positions were projected onto the scanning plane with a Gaussian filter and pixel noise applied to visually match resolution and noise of the scan-coil calibration and drift corrected experimental images. Red/green superpositions of the experimental and simulated structure FFTs (Figure S8C1-6) demonstrate that the crystalline Ru nanoclusters on GNF imaged along one of their zone-axes exhibit a Ru hcp lattice structure.

S6 DFT calculation

Density Functional Theory (DFT) calculations were performed using the plane wave code CASTEP[3]. The exchange and correlation interactions were modelled via the corrected density functional of Perdew, Burke and Ernzerhof (PBE)[4] within the generalized gradient approximation (GGA)[5], using non-conserving pseudopotentials (NCP). The kinetic energy cut-off of the plane wave basis was set to 800 eV whilst the semi-empirical dispersion correction G06 - Grimme 2006[6] has been applied. The Ru_{50} cluster was constructed based on the hcp crystal structure using three layers of Ru atoms. For the supported Ru_{50} cluster on graphene, a cell of 338 atoms has been used allowing approximately 8 Å vacuum around the cluster in each direction to avoid interactions with its periodic images. The system was allowed to relax until reaching the energetically favourable configuration corresponding to its optimized geometry whilst the cell parameters were kept fixed during relaxation.

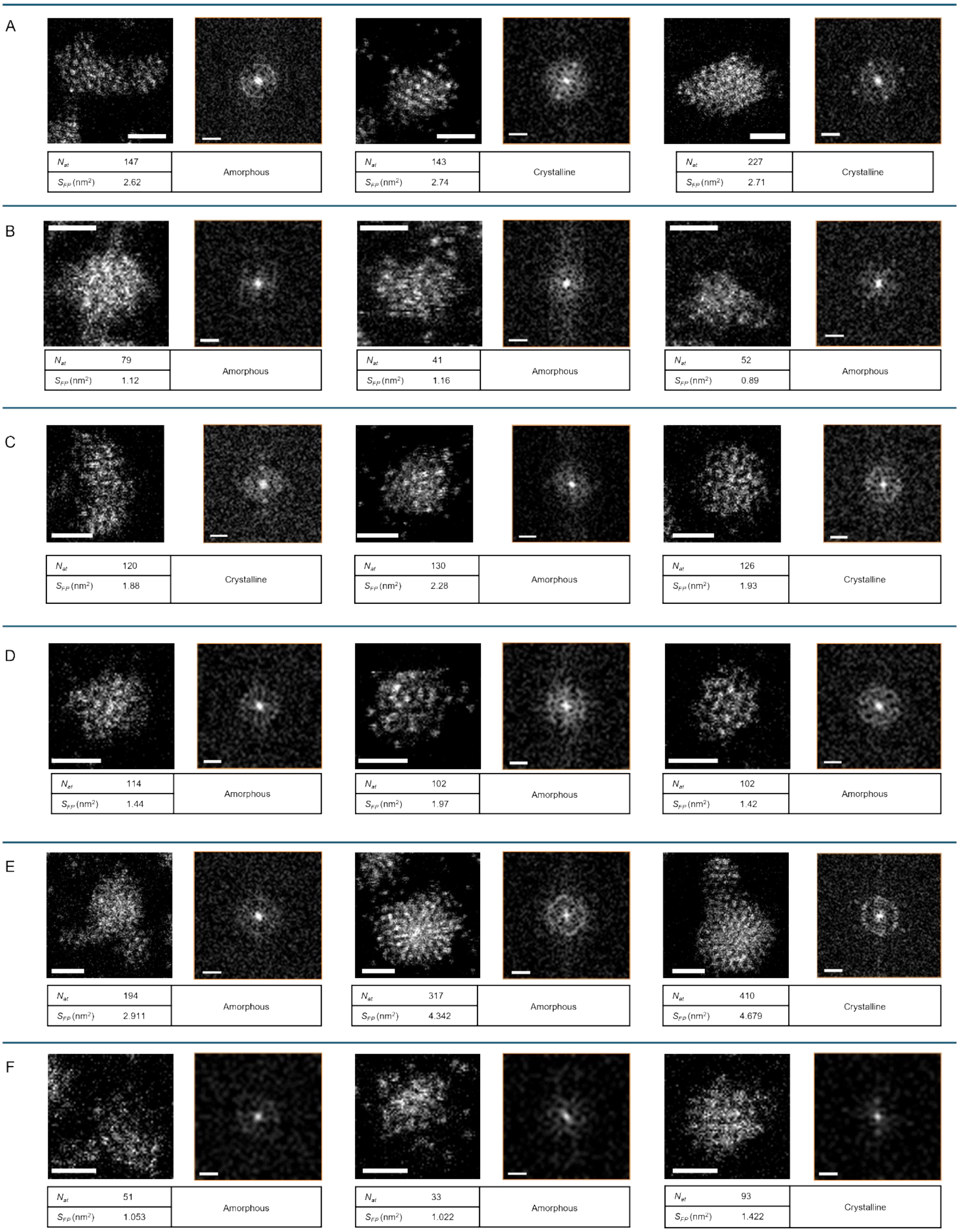

Figure S3: Evolution of Ru/GNF after H_2 treatment for 1 hour and NH_3 decomposition reaction for 3 hours: identical location observation of individual nanocluster. STEM images, FFT patterns is shown on the right side, and key nanocluster parameters are shown below each image. Scale bar in STEM image is 1 nm. Scale bar in FFT patterns is 5 1/nm.

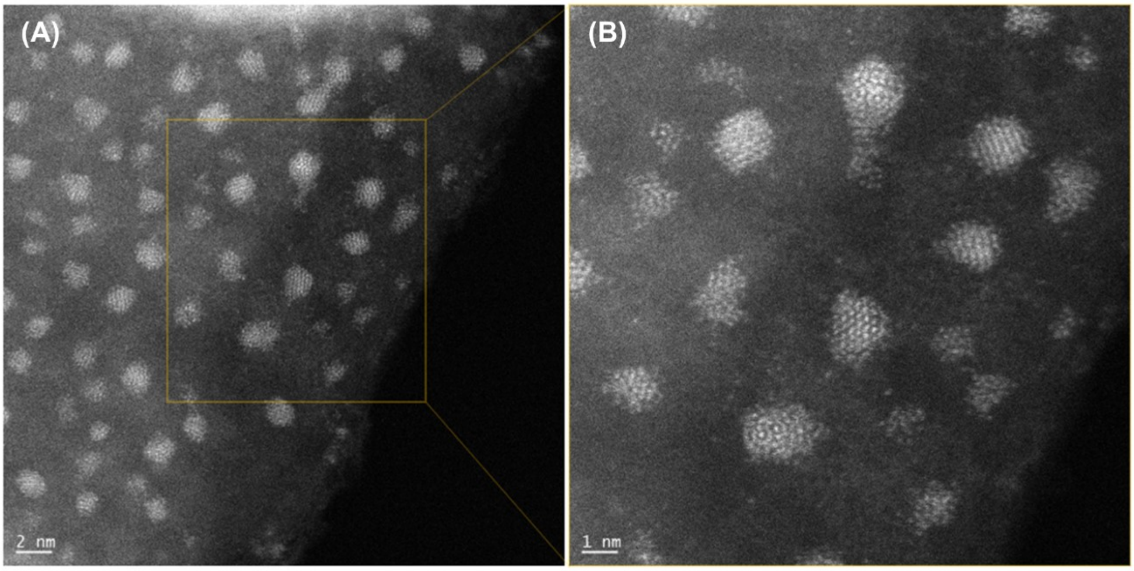

Figure S4: STEM images of Ru/GNF after NH_3 decomposition reaction for 1.5 hours.

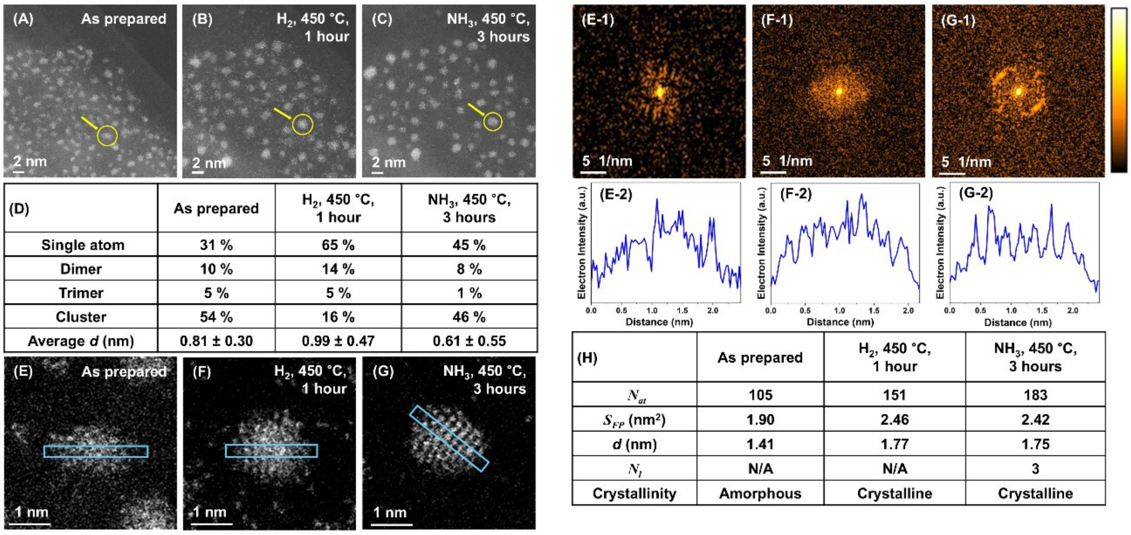

(D)	As prepared	H_2, 450 °C, 1 hour	NH_3, 450 °C, 3 hours
Single atom	31 %	65 %	45 %
Dimer	10 %	14 %	8 %
Trimer	5 %	5 %	1 %
Cluster	54 %	16 %	46 %
Average *d* (nm)	0.81 ± 0.30	0.99 ± 0.47	0.61 ± 0.55

(H)	As prepared	H_2, 450 °C, 1 hour	NH_3, 450 °C, 3 hours
N_{at}	105	151	183
S_{FP} (nm^2)	1.90	2.46	2.42
d (nm)	1.41	1.77	1.75
N_l	N/A	N/A	3
Crystallinity	Amorphous	Crystalline	Crystalline

Figure S5: IL-STEM images of Ru/GNF at different stages: as-prepared (A), after 450 °C in H_2 (B), and after 450 °C in NH_3 (C). A tabulated summary of changes in the population of single atoms, dimers, trimers and nanoclusters, and the nanoclusters' average *d* for each stage (D). An example of IL-STEM analysis for the evolution of a specific single Ru nanocluster (marked with the arrow in A-C) through different reaction stages (E-G), with corresponding FFT patterns (E1-G1) and intensity line profiles (E2-G2) cut along the directions marked on STEM images. A summary of key structural parameters for the single Ru nanocluster at different reaction stages (H), where N/A means not applicable.

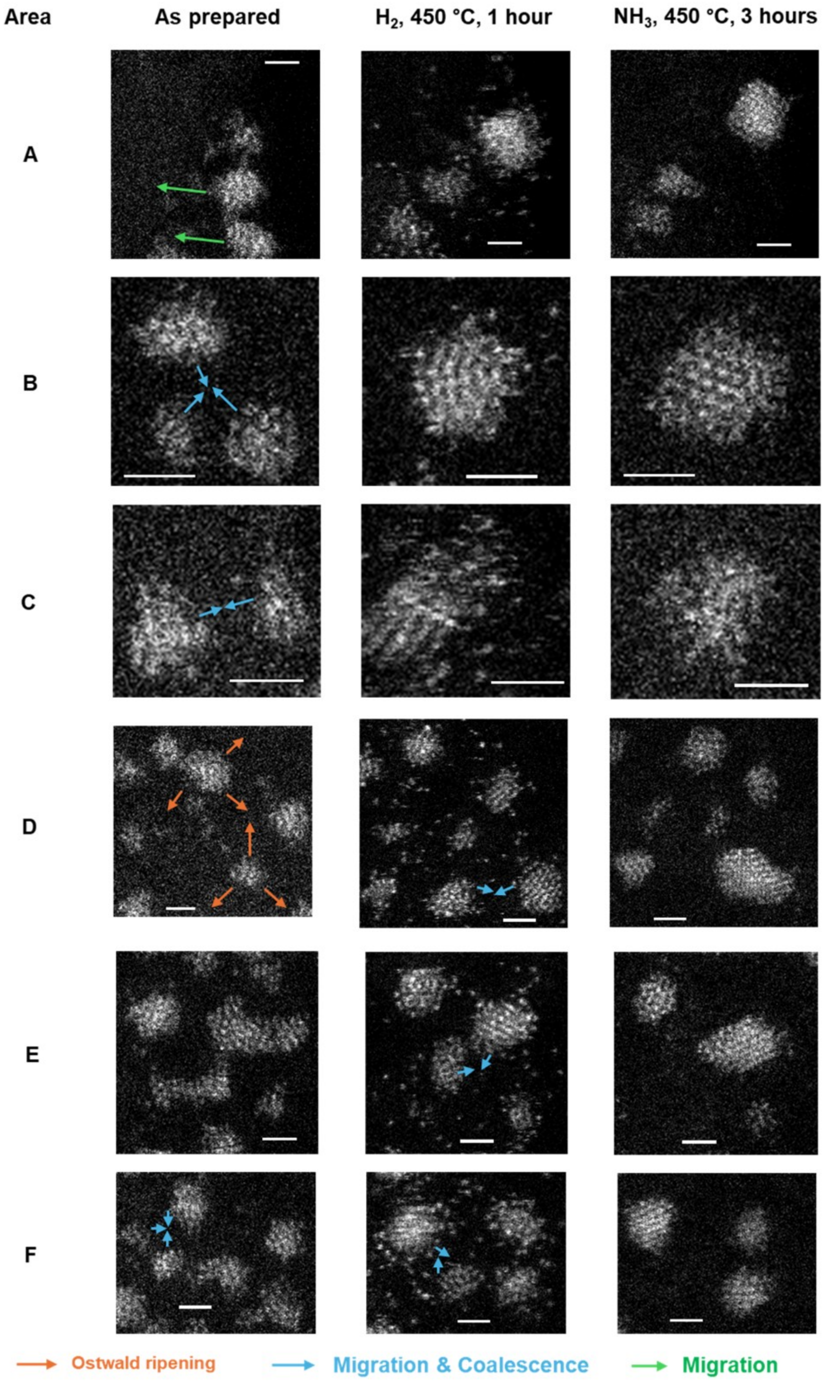

Figure S6: Evolution of groups of nanoclusters after H_2 treatment for 1 hour (middle column) and NH_3 decomposition reaction for 3 hours (right column): identical location observation. Changes in nanoclusters are indicated by arrows (orange = Ostwald ripening; blue = migration and coalescence; green = migration without coalescence). Scale bar, 1 nm. Large field-of-view STEM images from which these individual events were taken are shown in the main manuscript, Figures 3A, B and C.

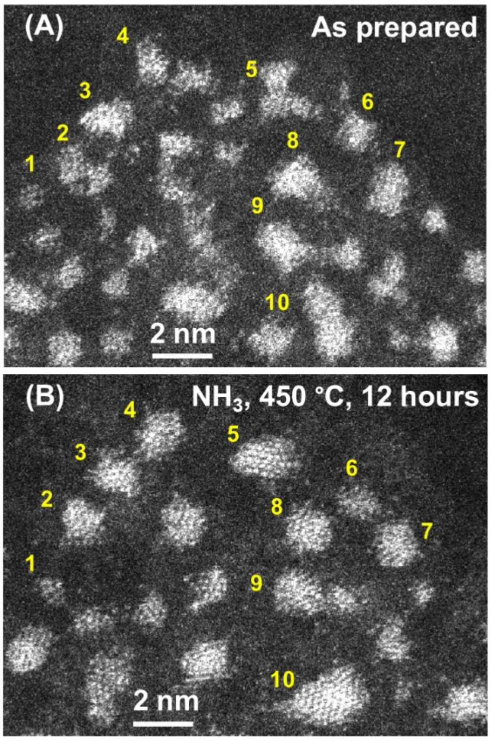

(C) N_{at} evolution		
Position	As prepared	NH_3, 450 °C, 12 hours
1	16	25
2	109	143
3	153	136
4	142	117
5	138	187
6	73	36
7	106	123
8	148	166
9	145	153
10	162	374

Figure S7: IL-STEM images of Ru/GNF at different stages: as-prepared (A) and after 450 °C in NH_3 for 12 hours (B). A tabulated summary of changes in the N_{at} for each nanocluster marked in A and B (C).

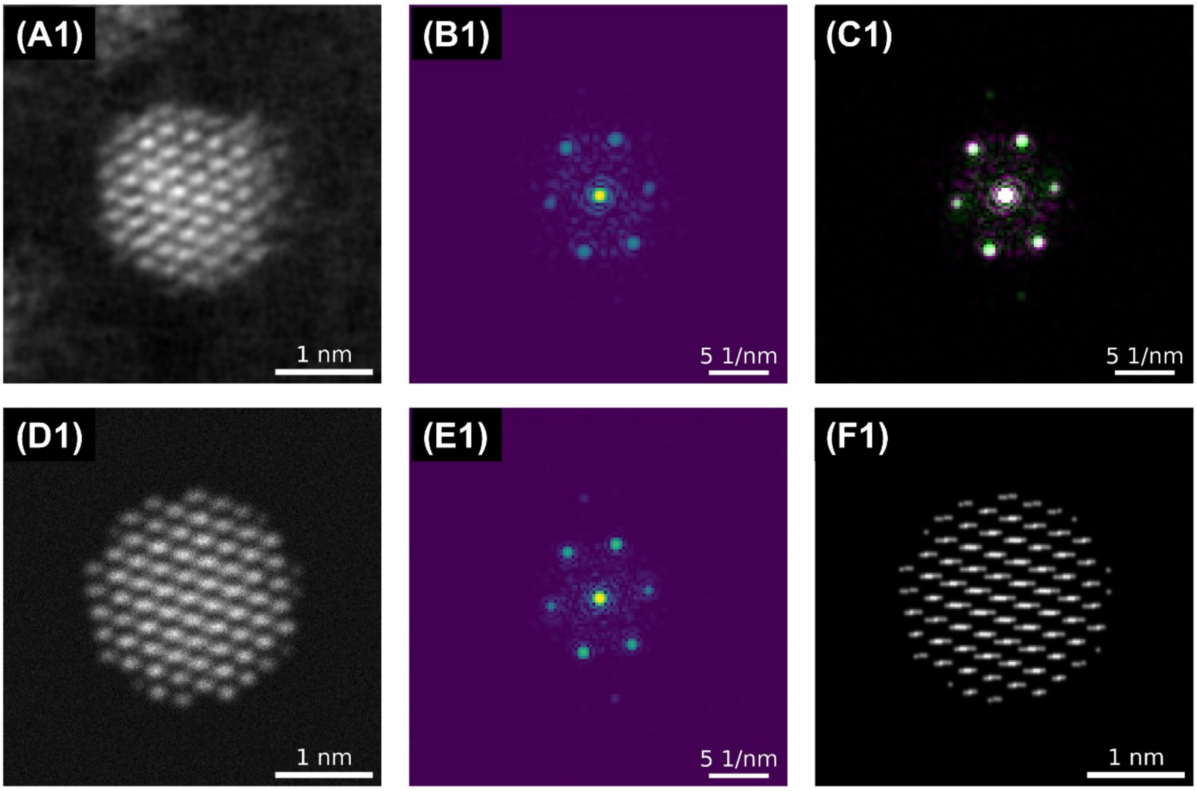
(A1)
1 nm
(B1)
5 1/nm
(C1)
5 1/nm
(D1)
1 nm
(E1)
5 1/nm
(F1)
1 nm

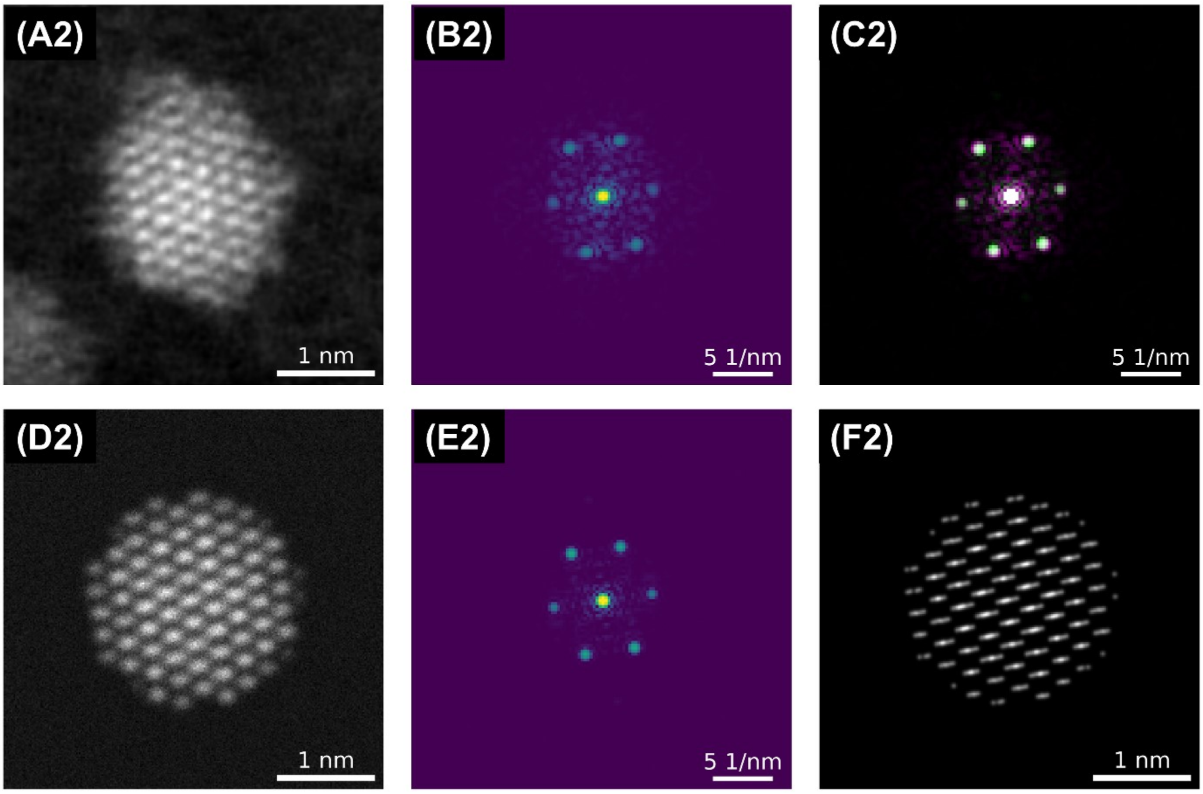
(A2)
1 nm
(B2)
5 1/nm
(C2)
5 1/nm
(D2)
1 nm
(E2)
5 1/nm
(F2)
1 nm

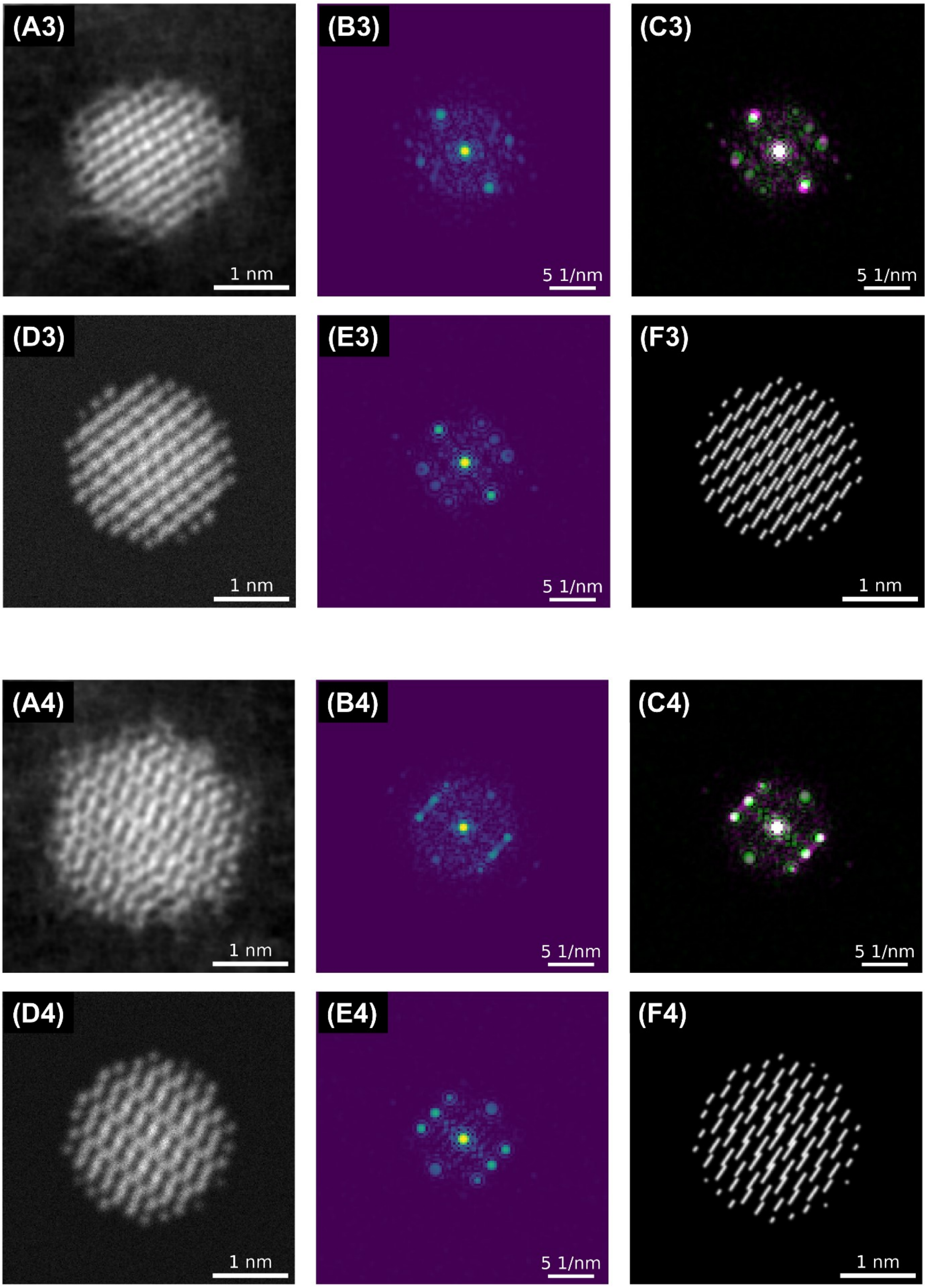
(A3)
1 nm
(B3)
5 1/nm
(C3)
5 1/nm
(D3)
1 nm
(E3)
5 1/nm
(F3)
1 nm
(A4)
1 nm
(B4)
5 1/nm
(C4)
5 1/nm
(D4)
1 nm
(E4)
5 1/nm
(F4)
1 nm

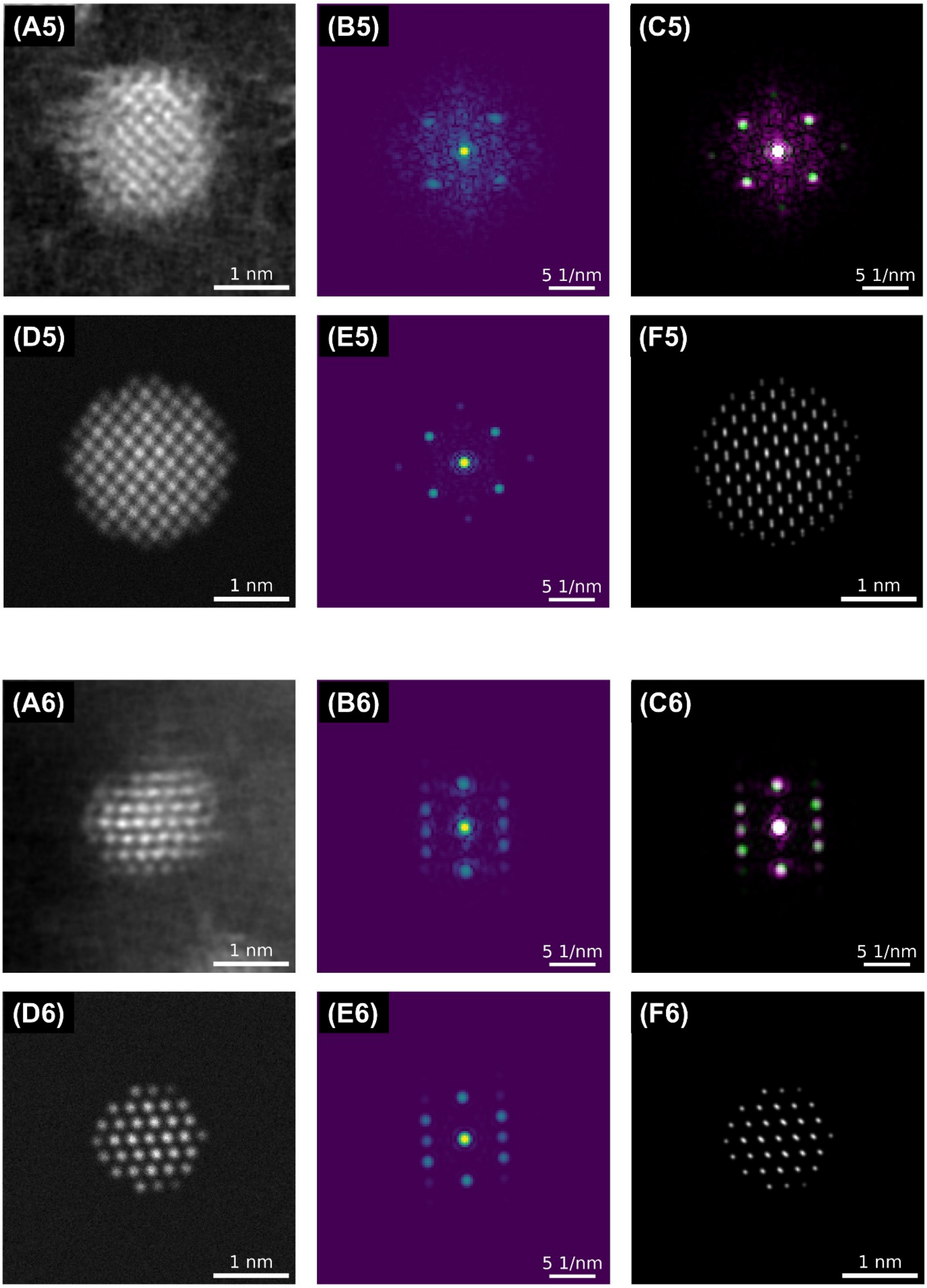

Figure S8: Comparison of experimental and simulated AC-STEM microscopy data for six nanoclusters to elucidate the selected nanoclusters' lattice structure. (A) Drift and scancoil

calibration corrected experimental image derived from a 0/90 degree scan rotation image pair and corresponding FFT (B). (D) Simulated AC-STEM image of a suitably rotated spherical nanocluster with Ru hcp lattice and Ru bulk lattice constant, and its FFT (E). (C) Red/green channel superposition of (B) and (C), respectively. (F) Version of (D) with minimal broadening and without noise to visualise the projected atomic positions.

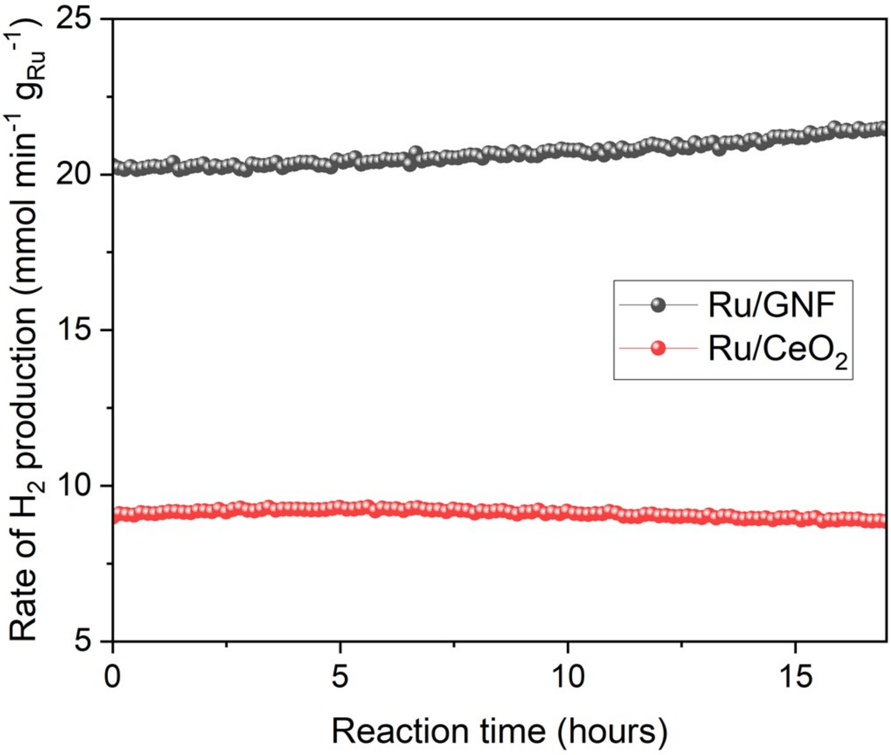

Figure S9: Ru/GNF and Ru/CeO_2 catalytic activity comparison for H_2 production from ammonia decomposition.

Table S2. Comparison of rate of hydrogen production and turn over frequency (TOF) of our catalyst with literature catalysts.

Catalyst	GHSV (mL gcat^{-1} h^{-1})	Temperature (°C)	Rate of H_2 production (mmol g_{Ru}^{-1} min^{-1})	TOF (h^{-1})	Reference
**Ru/GNF**	**30000**	**450**	**27**	**170**	**This work**
Ru/C	32000	450	40	-	[7]
RuP2D			40		
Ru-V2d			95		
1Ru/20C-rGO	150000	400	-	78	[8]
2.5Ru/20C-rGO				63	
5Ru/20C-rGO				70	
Ru/ CNT	6000	400		183	[9]
Ru/CNTs	60000	400	119	-	[10]
Ru-Mg(NH2)2			24		
Ru/SiC	60000	400	83	-	[11]
Ru/CNTs	30000	450	-	265	[12]
Ru/AC				113	
RU/Al_2O_3				240	
Ru/MgO				252	
Ru/TiO_2				214	

1. https://github.com/ptim0626/scanning-drift-corr (release 1.0.1)).
2. C. Ophus, J. Ciston and C. T. Nelson, Correcting nonlinear drift distortion of scanning probe and scanning transmission electron microscopies from image pairs with orthogonal scan directions, *Ultramicroscopy*, 2016, **162**, 1-9.
3. M. D. Segall, J. D. L. Philip, M. J. Probert, C. J. Pickard, P. J. Hasnip, S. J. Clark and M. C. Payne, First-principles simulation: ideas, illustrations and the CASTEP code, *Journal of Physics: Condensed Matter*, 2002, **14**, 2717.
4. J. P. Perdew, K. Burke and M. Ernzerhof, Generalized Gradient Approximation Made Simple, *Physical Review Letters*, 1996, **77**, 3865-3868.
5. D. Vanderbilt, Soft self-consistent pseudopotentials in a generalized eigenvalue formalism, *Physical Review B*, 1990, **41**, 7892-7895.
6. S. Grimme, Semiempirical GGA-type density functional constructed with a long-range dispersion correction, *Journal of Computational Chemistry*, 2006, **27**, 1787-1799.
7. S.-J. Kim, T. S. Nguyen, J. Mahmood and C. T. Yavuz, Sintering-free catalytic ammonia cracking by vertically standing 2D porous framework supported Ru nanocatalysts, *Chemical Engineering Journal*, 2023, **463**, 142474.
8. M. Pinzón, O. Avilés-García, A. R. de la Osa, A. de Lucas-Consuegra, P. Sánchez and A. Romero, New catalysts based on reduced graphene oxide for hydrogen production from ammonia decomposition, *Sustainable Chemistry and Pharmacy*, 2022, **25**, 100615.
9. T. E. Bell, G. Zhan, K. Wu, H. C. Zeng and L. Torrente-Murciano, Modification of Ammonia Decomposition Activity of Ruthenium Nanoparticles by N-Doping of CNT Supports, *Topics in Catalysis*, 2017, **60**, 1251-1259.
10. P. Yu, J. Guo, L. Liu, P. Wang, F. Chang, H. Wang, X. Ju and P. Chen, Effects of Alkaline Earth Metal Amides on Ru in Catalytic Ammonia Decomposition, *The Journal of Physical Chemistry C*, 2016, **120**, 2822-2828.
11. M. Pinzón, A. Romero, A. de Lucas Consuegra, A. R. de la Osa and P. Sánchez, Hydrogen production by ammonia decomposition over ruthenium supported on SiC catalyst, *Journal of Industrial and Engineering Chemistry*, 2021, **94**, 326-335.
12. S. F. Yin, B. Q. Xu, W. X. Zhu, C. F. Ng, X. P. Zhou and C. T. Au, Carbon nanotubes-supported Ru catalyst for the generation of COx-free hydrogen from ammonia, *Catalysis Today*, 2004, **93-95**, 27-38.
